# Long non-coding RNA AFAP1-AS1 facilitates ovarian cancer progression by regulating the miR-107/PDK4 axis

**DOI:** 10.1186/s13048-021-00808-x

**Published:** 2021-04-29

**Authors:** Bao Liu, Li Yan, Yugang Chi, Yuhan Sun, Xiaoyu Yang

**Affiliations:** Chongqing Health Center for Women and Children, Chongqing, 401147 China

**Keywords:** AFAP1-AS1, Ovarian cancer (OC), MiR-107, PDK4

## Abstract

**Background:**

Abnormally expressed in various tumors, long non-coding RNAs (lncRNAs) feature prominently in tumor development, yet little is still known regarding the functional roles of lncRNA AFAP1 antisense RNA 1 (AFAP1-AS1) in ovarian cancer (OC).

**Methods:**

The relative expression levels of lncRNA AFAP1-AS1, microRNA (miR)-107 and pyruvate dehydrogenase kinase isozyme 4 (PDK4) mRNA were assessed by quantitative real-time PCR. PDK4, PCNA and cyclin D1 expression levels were determined using Western blot analysis. Bioinformatics analysis and dual-luciferase gene reporter assay were conducted for identifying and validating the binding sequences between AFAP1-AS1 and miR-107, as well as between miR-107 and PDK4. Cell counting kit-8 assay was employed for detecting cell proliferation. Cell migration and invasion abilities were examined using Transwell assays.

**Results:**

The present study revealed that AFAP1-AS1 expression was elevated in OC cells and tissues. AFAP1-AS1 expression and FIGO stage were positively correlated. AFAP1-AS1 knockdown repressed OC cell proliferation, migration and invasion. AFAP1-AS1 functioned as a sponge of miR-107, and miR-107 reversed the effects of AFAP1-AS1 on OC cells. It was validated that miR-107 was able to bind to PDK4, and AFAP1-AS1 regulated PDK4 expression by competitively binding with miR-107. Additionally, miR-107 modulated OC cell proliferation, migration and invasion via targeting PDK4.

**Conclusions:**

LncRNA AFAP1-AS1 serves as a tumor driver in the pathogenesis of OC via the miR-107/PDK4 axis.

## Introduction

Ovarian cancer (OC) is the second most common cause of cancer-related deaths among women [1]. Statistically, there were a total of 22,240 new cases and 14,070 death cases worldwide in 2018 [1]. Since the early symptoms of OC are insidious and the screening methods are unreliable, most of the patients are not diagnosed until they reach the advanced stage [2, 3]. Although surgery and chemotherapy can improve the survival, the five-year survival rate of OC patients remains very low [2, 3]. Therefore, exploring the molecular mechanism of OC occurrence and development is highly significant for the early diagnosis, prognosis judgment and individualized targeted therapy of OC.

Recognized as a class of functional RNA molecule whose transcripts are over 200 *nt* long, long non-coding RNAs (lncRNAs) modulate gene expression at the epigenetic, transcriptional and post-transcriptional levels, thereby participating widely in the physiological and pathological processes [4, 5]. Although lncRNAs lack the ability to encode proteins, they can still serve as tumor-suppressing genes or oncogenes, and can function as potential diagnostic and prognostic biomarkers [6–8]. LncRNA AFAP1 antisense RNA 1 (AFAP1-AS1) was first found in 2013 in esophageal adenocarcinoma [9]. Accumulating evidence shows that AFAP1-AS1 plays a cancer-promoting role in the progression of various tumors. Reportedly, AFAP1-AS1 facilitates the tumorigenesis and epithelial-mesenchymal transition of triple-negative breast cancer via the Wnt/β-Catenin signal pathway [10]. AFAP1-AS1 facilitates non-small cell lung cancer cell migration and invasion by up-regulating the IRF7 and RIG-I-like receptor signaling pathways [11]. In addition, a study has found that AFAP1-AS1 is highly expressed in OC cells and tissues, and AFAP1-AS1 knockdown markedly suppresses the proliferation of OC cells and facilitates the apoptosis [12]. Nonetheless, the molecular mechanism of AFAP1-AS1 regulating OC cell behaviors is still unclear.

Identified as a class of endogenous non-coding RNAs with a length of 18–25 *nt*, microRNAs (miRs or miRNAs) can bind specifically to the target mRNA 3′-untranslated region (3′-UTR) to block mRNA translation or induce its degradation, thus participating in modulating gene expression at the post-transcriptional level, and therefore modulating the pathogenesis of various human diseases including cancer [13, 14]. It is reported that miR-107 plays both cancer-promoting and cancer-suppressing roles in different tumors [15–17]. For instance, the significant up-regulation of miR-107 expression can be observed in gastric cancer (GC) tissues and cell lines, and miR-107 promotes GC cell proliferation and represses the apoptosis through the targeted regulation of PTEN [15]; conversely, miR-107 expression is markedly reduced in OC cell lines and tissues, and through the targeted inhibition of CCNE1, miR-107 induces the G0/G1 arrest of OC cells, and significantly inhibits their colony-forming ability [17]. Nevertheless, the role and underlying mechanism of miR-107 in OC are yet to be well elaborated upon.

This research was performed to probe into the role and potential mechanism of AFAP1-AS1 in OC. It was revealed that AFAP1-AS1 regulated pyruvate dehydrogenase kinase isoform 4 (PDK4) expression by decoying miR-107, thereby promoting OC progression. Our study revealed that the AFAP1-AS1/miR-107/PDK4 axis in OC, and provided a novel mechanism explaining OC development.

## Materials and methods

### Clinical specimens

A total of 39 pairs OC tissues / adjacent tissues were collected from patients in Chongqing Health Center for Women and Children, and the samples were verified by two independent pathologists. Before the surgery, none of the patients underwent radiotherapy or chemotherapy. Signed informed consents were obtained from all patients. This study was approved by the Ethics Committee of Chongqing Health Center for Women and Children and followed the *Declaration of Helsinki*. All the tissue samples were preserved at − 80 °C until RNA extraction.

### Cell culture

Normal ovarian surface epithelial cell line (IOSE80) and human OC cell lines (COV504, OVISE, OV90 and SKOV3) were obtained from the American Type Culture Collection (Manassas, VA, USA). All the cells were cultured in high-glucose (4.5 mg/ml) DMEM (Hyclone, Logan, UT, USA) containing 10% fetal bovine serum (FBS; Hyclone, Logan, UT, USA), 100 U/ml streptomycin (Invitrogen, Carlsbad, CA, USA) and 100 mg/ml penicillin (Invitrogen, Carlsbad, CA, USA) at 37 °C in 5% CO_2_.

### Cell transfection

The miR-107 mimic (miR-107, 5′-AGCAGCAUUGUACAGGGCUAUCA-3′), negative control of miRNA mimic (miR-NC, 5′-UUCUUCGAAGGUGUGACAC-3′), miR-107 inhibitor (miR-107-in, 5′-UGAUAGCCCUGUACAAUGCUGCU-3′) and inhibitor control (miR-in, 5′-CAGUACUUUUGUGUAGUACA-3′), AFAP1-AS1 overexpression vector pcDNA3.1-AFAP1-AS1 (AFAP1-AS1), the empty vector pcDNA3.1 (Vector), small interfering RNAs against AFAP1-AS1 (si-AFAP1-AS1) (si-AFAP1-AS1#1, 5′-GGACCACUUUGGUGUAUCUTT-3′; si-AFAP1-AS1#2, 5′-GGGCUUCAAUUUACAAGCATT-3′) and their negative control (si-NC, 5′-UUCUCCGAACGUGUCACGUTT-3′) were designed and synthesized by GenePharma (Shanghai, China). GenePharma also synthesized the full-length sequence of PDK4 lacking the 3′-UTR, and the sequence was inserted into the pcDNA3.1 vector to produce the pcDNA3.1-PDK4 plasmid (PDK4). According to the manufacturer’s protocol, Lipofectamine 2000 (Invitrogen, Carlsbad, CA, USA) was employed to conduct cell transfection.

### Quantitative real-time PCR (qRT-PCR)

Following the manufacturer’s instructions, TRIzol reagent (TaKaRa, Dalian, China) was utilized for extracting total RNA. Then, PrimeScript RT Reagent Kit (TaKaRa, Dalian, China) was employed for reverse transcribing the RNA into cDNA. The qRT-PCR assay was conducted with SYBR Green Master Mix (TaKaRa, Dalian, China). GAPDH and U6 were used as the internal references for AFAP1-AS1, miR-107 and PDK4 mRNA, respectively. The 2^−ΔΔCt^ method was adopted for data analysis. Below are the primer sequences: AFAP1-AS1, 5′-CACACAGGGGAATGAAGAGG-3′ and 5′-AATGGTGGTAGGAGGGAGGA-3′; PDK4, 5′-GGAGCATTTCTCGCGCTACA-3′ and 5′-ACAGGCAATTCTTGTCGCAAA-3′; GAPDH, 5′-CATGAGAAGTATGACAACAGCCT-3′ and 5′-AGTCCTTCCACGATACCAAAGT-3′; miR-107, 5′-AGCAGCAUUGUACAGGGCUAUCA-3′; U6, 5′-CGCAAGGATGACACGCAAATTC-3′.

### Cell Counting Kit-8 (CCK-8) assay

According to the manufacturer’s protocol, the CCK-8 assay was employed for detecting cell proliferation. In brief, 2000 OC cells were transferred into each well of a 96-well plate. Ten microlitre of CCK-8 solution (Biotechwell, Shanghai, China) was added to each well at different time points (12, 24, 48, 72 and 96 h). Next, the cells were incubated with CCK-8 solution for 4 h. Then, the absorbance values of the wells at OD 450 nm at different time points were measured with a microplate reader.

### Transwell assay

Transwell chamber (Costar, Cambridge, MA, USA) with Matrigel (BD Biosciences, Franklin Lakes, NJ, USA) was utilized for detecting cell invasion. Transwell chamber without Matrigel was used for detecting cell migration. 1 × 10^5^ cells were added into the upper chamber with serum-free medium. DMEM with 10% FBS was added to the lower chamber. Then the cells were placed in the incubator, and cultured for 24 h. Next, the cells on the upper surface of the membrane were removed. Subsequently, the cells on the below surface of the membrane were fixed with 90% ethyl alcohol and stained for 30 min with 0.1% crystal violet solution. At last, a microscope was employed for counting the number of migrated and invaded cells.

### Dual-luciferase reporter assay

The sequences of AFAP1-AS1 or PDK4 3′-UTR containing wild-type (WT) or mutant (MUT) binding sites of miR-107 were cloned into the pmirGLO vector (Promega, Madison, WI, USA) to form AFAP1-AS1-WT, AFAP1-AS1-MUT, PDK4-WT or PDK4-MUT, respectively. Next, COV504 and SKOV3 cells were co-transfected with the corresponding luciferase reporter and miR-107 or miR-NC. Then the cells were cultured for 48 h, and then a Dual-Luciferase Assay Kit (Solarbio, Beijin, China) was adopted to examine the luciferase activity of the cells in each group.

### RNA immunoprecipitation (RIP) assay

Following the manufacturer’s instructions, Magna RIP kit (Millipore, Billerica, MA, USA) was utilized for conducting RIP assay. Cell lysate (from OC cells) was mixed with RIP buffer containing magnetic beads coated with human IgG or Ago2 antibody (Millipore, Billerica, MA, USA). Proteinase K was used to remove the proteins from immunoprecipitated RNA. Eventually, purified RNA was obtained, and the enrichment of AFAP-AS1 and miR-107 was detected through qRT-PCR.

### Western blot assay

RIPA lysis buffer (Beyotime, Shanghai, China) with proteinase inhibitor was utilized for extracting proteins from OC cells. A bicinchoninic acid protein assay kit (Applygen Technologies, Inc., Beijing, China) was employed for quantifying protein concentrations. Equal amounts of protein (20 μg) from each sample were loaded on a 10% SDS gel and resolved by SDS-PAGE and transferred to the PVDF membrane (EMD Millipore, Burlington, MA, USA). The membranes were blocked with 5% non-fat milk at room temperature for 1 h and subsequently incubated overnight at 4 °C with primary antibodies: anti-PDK4 antibody (1:500 dilution; ab214938, Abcam, Shanghai, China), anti-cyclin D1 antibody (1:500 dilution; ab40754, Abcam, Shanghai, China), anti-PCNA antibody (1:500 dilution; ab92552, Abcam, Shanghai, China) and anti-GAPDH antibody (1:1000 dilution; ab181602, Abcam, Shanghai, China). After being washed with PBS, the membranes were incubated with the corresponding goat anti-rabbit IgG H&L secondary antibodies (1:2000 dilution; Abcam, Shanghai, China) for 1 h at room temperature. The protein bands were visualized using the Enhanced Chemiluminescence Detection Kit (Promega, Madison, WI, USA), and quantified with Quantity One software (Bio-Rad, Hercules, CA, USA).

### Statistical analysis

SPSS v22.0 (SPSS, Inc., Chicago, IL, USA) was used for statistical analysis. Each experiment was conducted at least in triplicate. All measurement data were shown as “mean ± standard deviation”. The comparison between two groups and among multiple groups were conducted with student’s *t*-test and one-way analysis of variance (ANOVA), respectively. Chi-square test was conducted for the analysis of the count data. Survival curves were generated using the Kaplan-Meier method and analyzed by log-rank test. A difference was considered to be of statistical significance if *P* < 0.05.

## Results

### AFAP1-AS1 was highly expressed and was associated with poor prognosis in OC

**Fig. 1:**
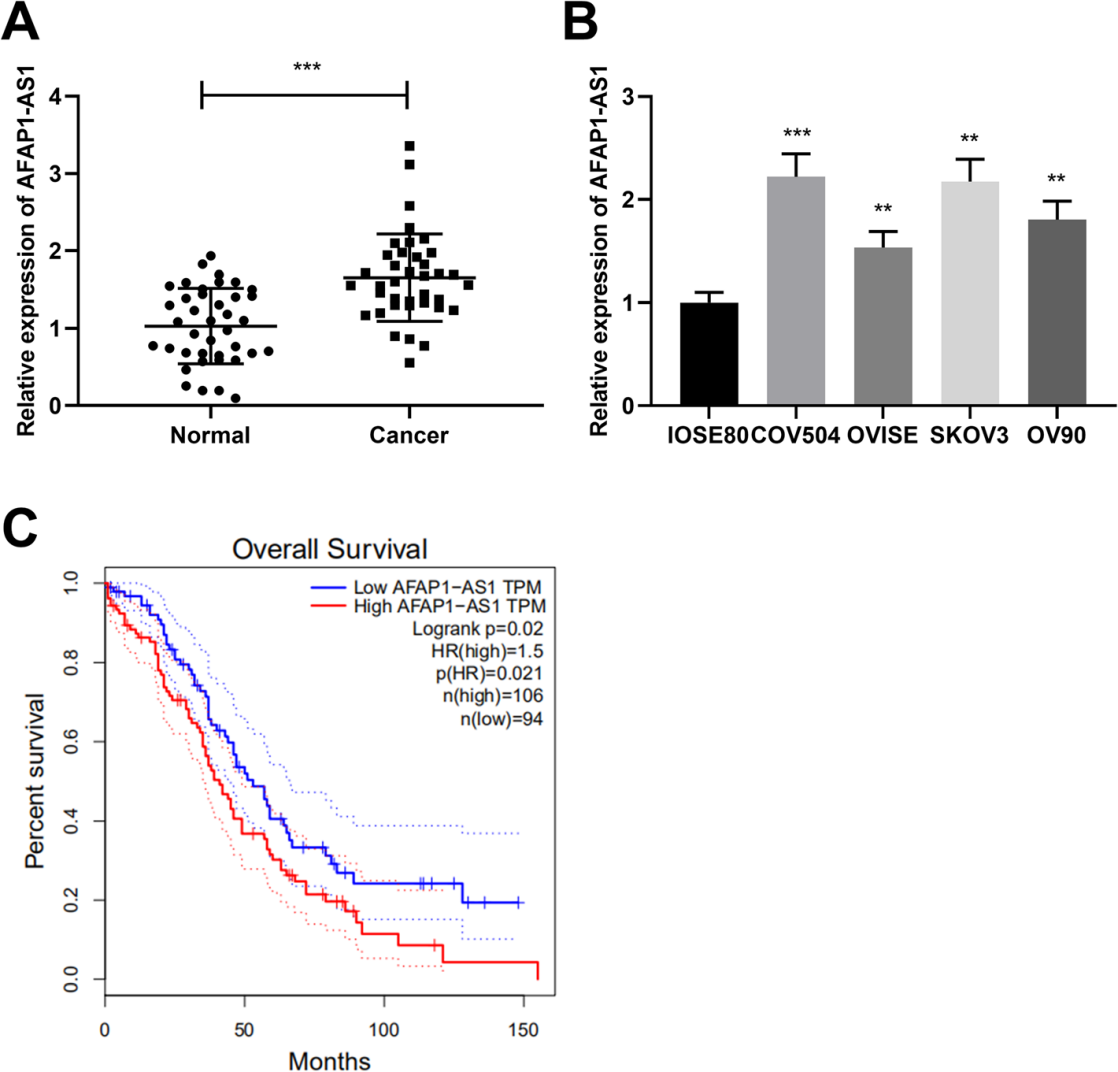
AFAP1-AS1 was highly expressed in OC. **a**. qRT-PCR was used for detecting AFAP1-AS1 expression in OC tissues and adjacent tissues. **b**. qRT-PCR was performed for assessing AFAP1-AS1 expression in OC cells and IOSE80 cells. **c**. The GEPIA database was utilized for analyzing the correlation between AFAP1-AS1 expression and the OC patient’s prognosis. **P < 0.01 and ***P < 0.001

The qRT-PCR was used to detect AFAP1-AS1 expression in OC cells and tissues, and it was shown that in comparison with normal human ovarian surface epithelial cell line (IOSE80) or adjacent tissues, AFAP1-AS1 expression was dramatically enhanced in OC tissues and cell lines (Fig. 1a&b). Next, the data from the GEPIA database showed that OC patients with lower AFAP1-AS1 expression displayed greater survival probability (Fig. 1c). In addition, the Chi-square analysis manifested that AFAP1-AS1 expression was positively correlated with the OC patient’s FIGO stage (Table 1). The above-mentioned evidence manifested that AFAP1-AS1 was likely to play a cancer-promoting part in OC, and AFAP1-AS1 could function as a potential biomarker of the poor prognosis of OC patients.

**Table 1:** Correlation between AFAP1-AS1 expression and clinicopathological features of OC patients (*n* = 39)

Characteristics	Number	LINC00473 expression		***χ***^***2***^	***P*** value
		Low	High		
Age (years)					
≤ 50	23	11	12	0.0631	0.8017
> 50	16	7	9		
Lymph node metastasis					
Absent	18	10	8	1.1890	0.2755
Present	21	8	13		
FIGO stage					
I–II	15	11	4	7.2454	0.0071
III–IV	24	7	17		
Pathologic type					
Serous	22	9	13	0.5586	0.4548
Mucous and others	17	9	8		
Serum CA125 (U/mL)					
≤ 675	24	12	12	0.3714	0.5422
> 675	15	6	9		
Tumor size (cm)					
≤ 5	22	8	14	1.947	0.206
> 5	17	10	7		

### AFAP1-AS1 knockdown inhibited the proliferation, migration and invasion of OC cells

**Fig. 2:**
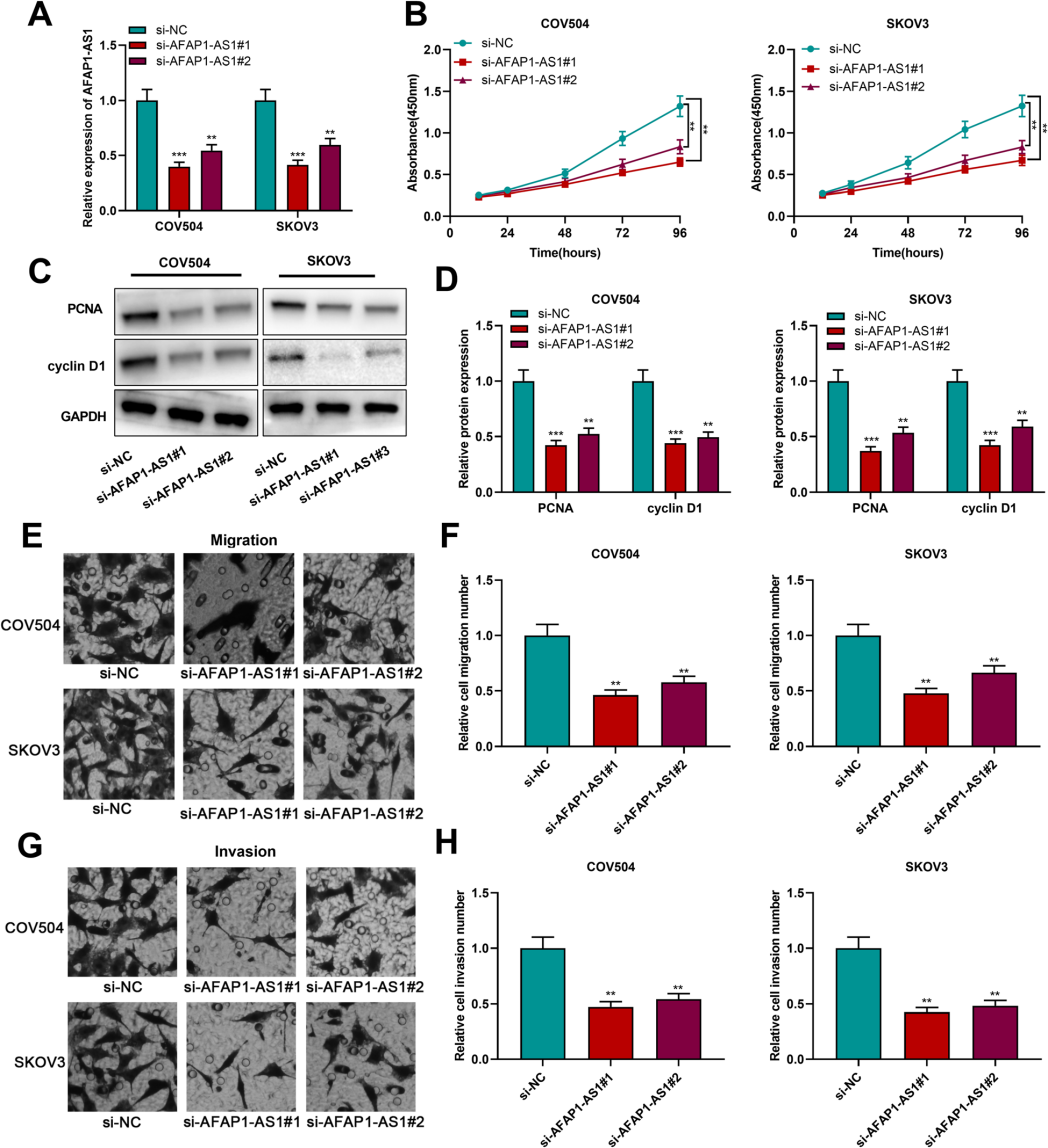
AFAP1-AS1 knockdown inhibited OC cell proliferation, migration and invasion. **a**. qRT-PCR was conducted for detecting AFAP1-AS1 expression in COV504 and SKOV3 cells after the transfection of AFAP1-AS1 siRNAs. **b**. CCK-8 method was employed for examining cell proliferation after AFAP1-AS1 knockdown. **c & d**. Western blot was used for detecting PCNA and cyclin D1 protein expression levels. **e-h**. Transwell assays were conducted for detecting cell migration and invasion. **P < 0.01 and ***P < 0.001

To probe into the biological functions of AFAP1-AS1 in OC progression, COV504 and SKOV3 cells with the higher AFAP1-AS1 expression were transfected with si-AFAP1-AS1 (si-AFAP1-AS1#1 and si-AFAP-1AS1#2) (Fig. 2a). CCK-8 assay results showed that, in comparison to the control group, AFAP1-AS1 knockdown remarkably suppressed OC cell proliferation (Fig. 2b). Additionally, Western blotting suggested that AFAP1-AS1 knockdown repressed the protein expression of cell proliferation-related genes [cyclin D1 and proliferating cell nuclear antigen (PCNA)] (Fig. 2c&d). Cell migration and invasion were detected by Transwell assays, and it was indicated that OC cell migration and invasion were inhibited due to AFAP1-AS1 knockdown (Fig. 2e-h).

### AFAP1-AS1 targeted miR-107

**Fig. 3:**
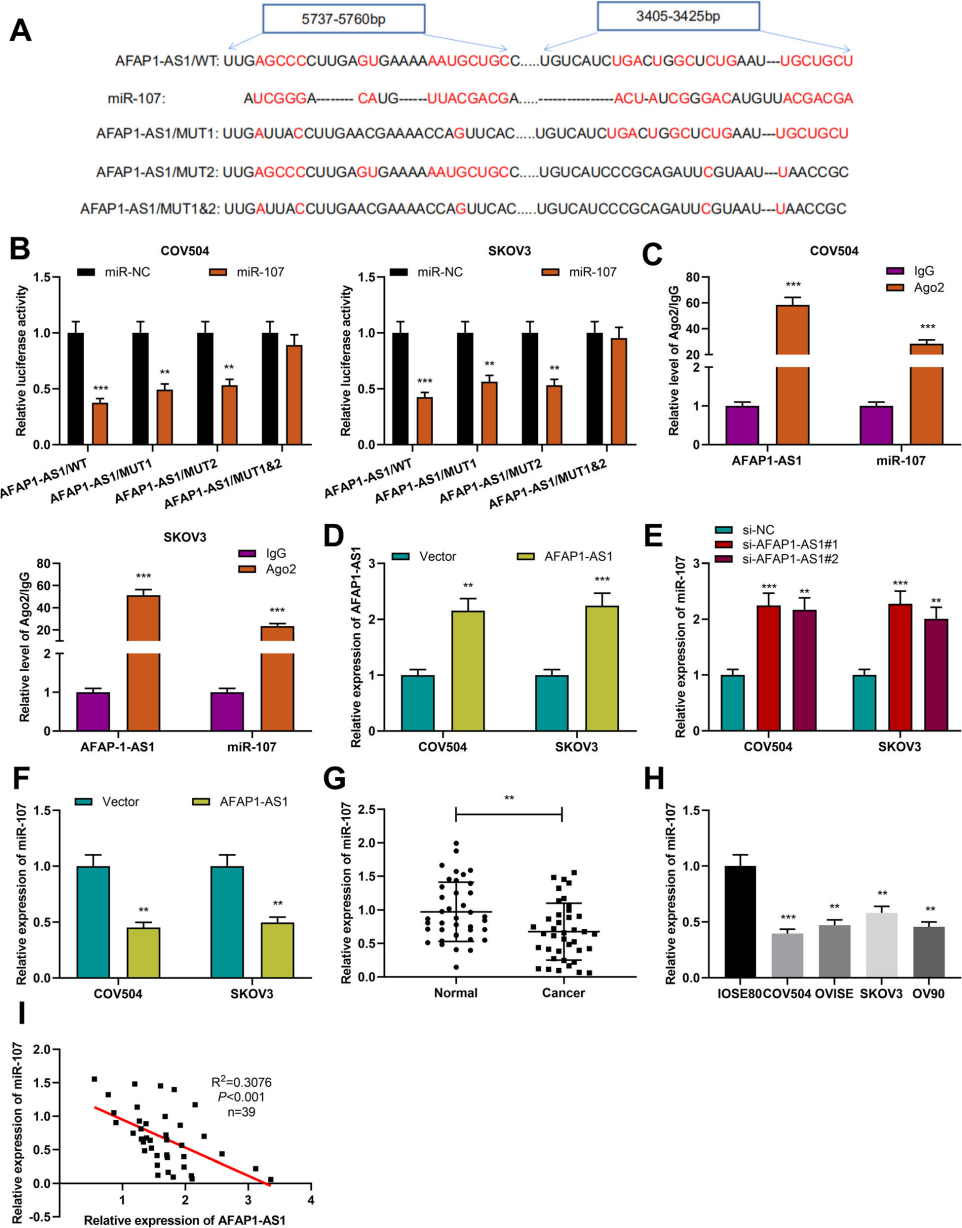
MiR-107 was the target of AFAP1-AS. **a**. Bioinformatics analysis predicted the binding sequences between miR-107 and AFAP1-AS1. **b**. Dual-luciferase reporter gene assay was utilized for verifying the binding relationship between miR-107 and AFAP1-AS1. **c**. RIP assay confirmed the relationship between AFAP1-AS1 and miR-107. **d**. qRT-PCR was performed for detecting AFAP1-AS1 expression in OC cells overexpressing AFAP1-AS1. **e & f**. qRT-PCR was conducted for examining miR-107 expression in cells with AFAP1-AS1 knockdown or overexpression. **g & h**. qRT-PCR was utilized for detecting miR-107 expression in OC tissues and cells. **i**. Correlation between miR-107 and AFAP1-AS1 expression levels in OC tissues. **P < 0.01 and ***P < 0.001

To make clear the molecular mechanism of AFAP1-AS1 regulating OC progression, the LncBase Predicted database was employed to predict the downstream targets of AFAP1-AS1, and it was found that miR-107 might be one of the functional targets of AFAP1-AS1 (Fig. 3a). To verify the targeted relationship between miR-107 and AFAP1-AS1, a dual-luciferase reporter gene assay was conducted, and it was demonstrated that miR-107 mimics notably inhibited the luciferase activity of AFAP1-AS1/WT, AFAP1-AS1/MUT1, AFAP1-AS1/MUT2, but exerted no significant impact on the luciferase activity of AFAP1-AS1/MUT1&2 (Fig. 3b). RIP assay results displayed that AFAP1-AS1 and miR-107 expression were enriched in the Ago2 pellet as opposed to the IgG control pellet (Fig. 3c). We further transfected AFAP1-AS1 overexpression plasmid into COV504 and SKOV3 cells (Fig. 3d). It was observed that miR-107 expression was increased in OC cells with AFAP1-AS1 knockdown, and AFAP1-AS1 overexpression significantly inhibited miR-107 expression in OC cells (Fig. 3e&f). In addition, we also examined miR-107 expression in OC. qRT-PCR indicated that miR-107 expression in OC cells and tissues was significantly reduced, and AFAP1-AS1 and miR-107 expression levels in OC tissues were negatively correlated (Fig. 3g-i). In conclusion, miR-107 was sponged by AFAP1-AS1 in OC, and miR-107 expression was negatively regulated by AFAP1-AS1.

### MiR-107 reversed the functions of AFAP1-AS1 in OC

**Fig. 4:**
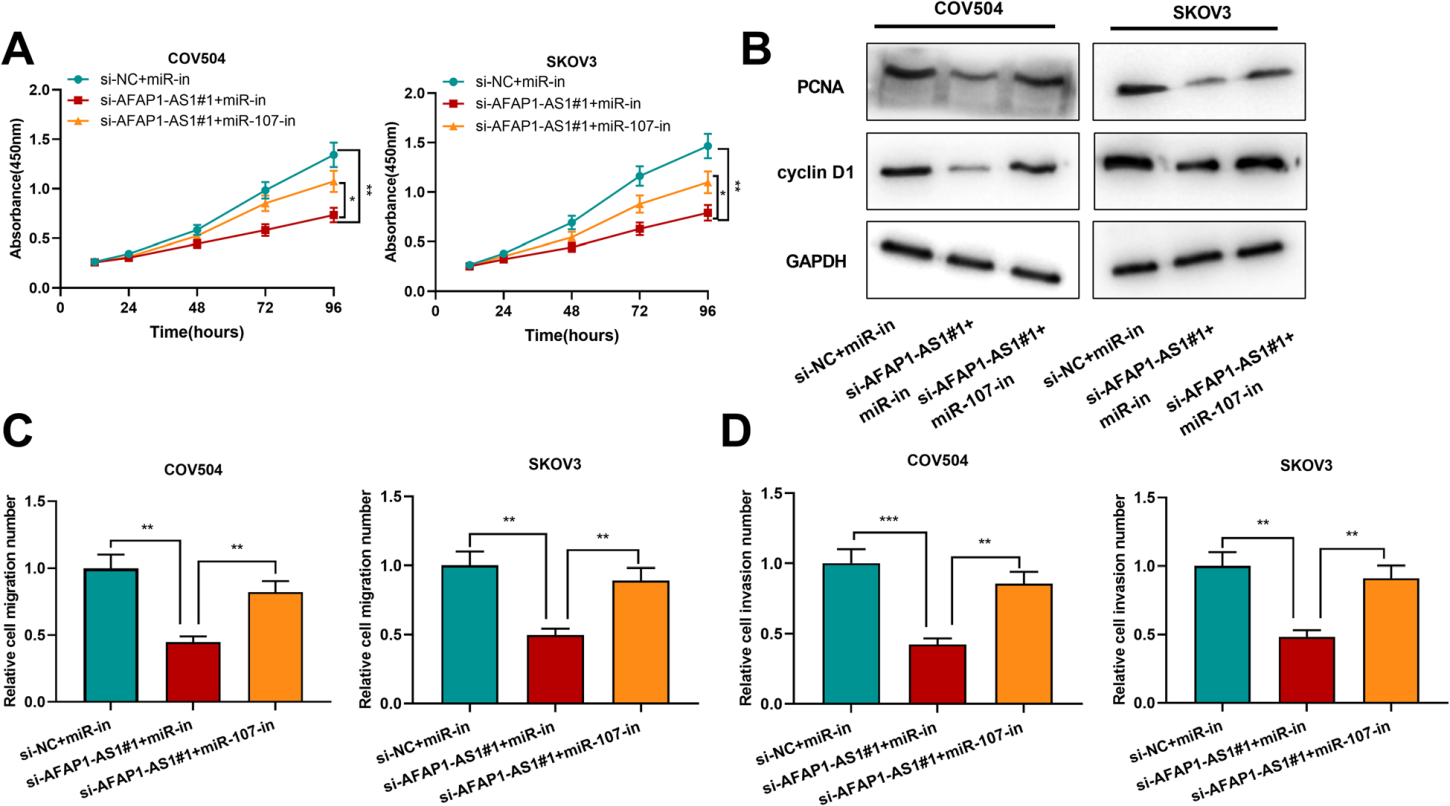
The AFAP1-AS1/miR-107 axis was involved in regulating OC cell proliferation, migration and invasion. **a**. AFAP1-AS1 siRNA and miR-107 were co-transfected into OC cells, and CCK-8 assay was conducted for detecting cell proliferation after transfection. **b**. Western blot was used to examine PCNA and cyclin D1 protein expression levels. **c & d**. Transwell assays were used to detect cell migration and invasion after transfection. *P < 0.05, **P < 0.01 and ***P < 0.001

The results mentioned above indicated that AFAP1-AS1 could negatively regulate miR-107. In the following experiments, the aim was to determine whether AFAP1-AS1 could modulate OC progression by targeting miR-107. First, the results of CCK-8 assay suggested that miR-107 inhibition reversed the inhibiting impact of AFAP1-AS1 knockdown on OC cell proliferation (Fig. 4a). Then, Western blotting was used for detecting PCNA and cyclin D1 expression levels, and it was revealed that miR-107 inhibition rescued the down-regulation of PCNA and cyclin D1 protein expression levels induced by AFAP1-AS1 knockdown in OC cells (Fig. 4b). Furthermore, the Transwell assays results indicated that the inhibiting effect of AFAP1-AS1 knockdown on OC cell migration and invasion was also significantly weakened by the transfection of miR-107-in (Fig. 4c&d). All the findings implied that miR-107 counteracted AFAP1-AS1-mediated proliferation, migration and invasion of OC cells.

### PDK4 was the direct target of miR-107

**Fig. 5:**
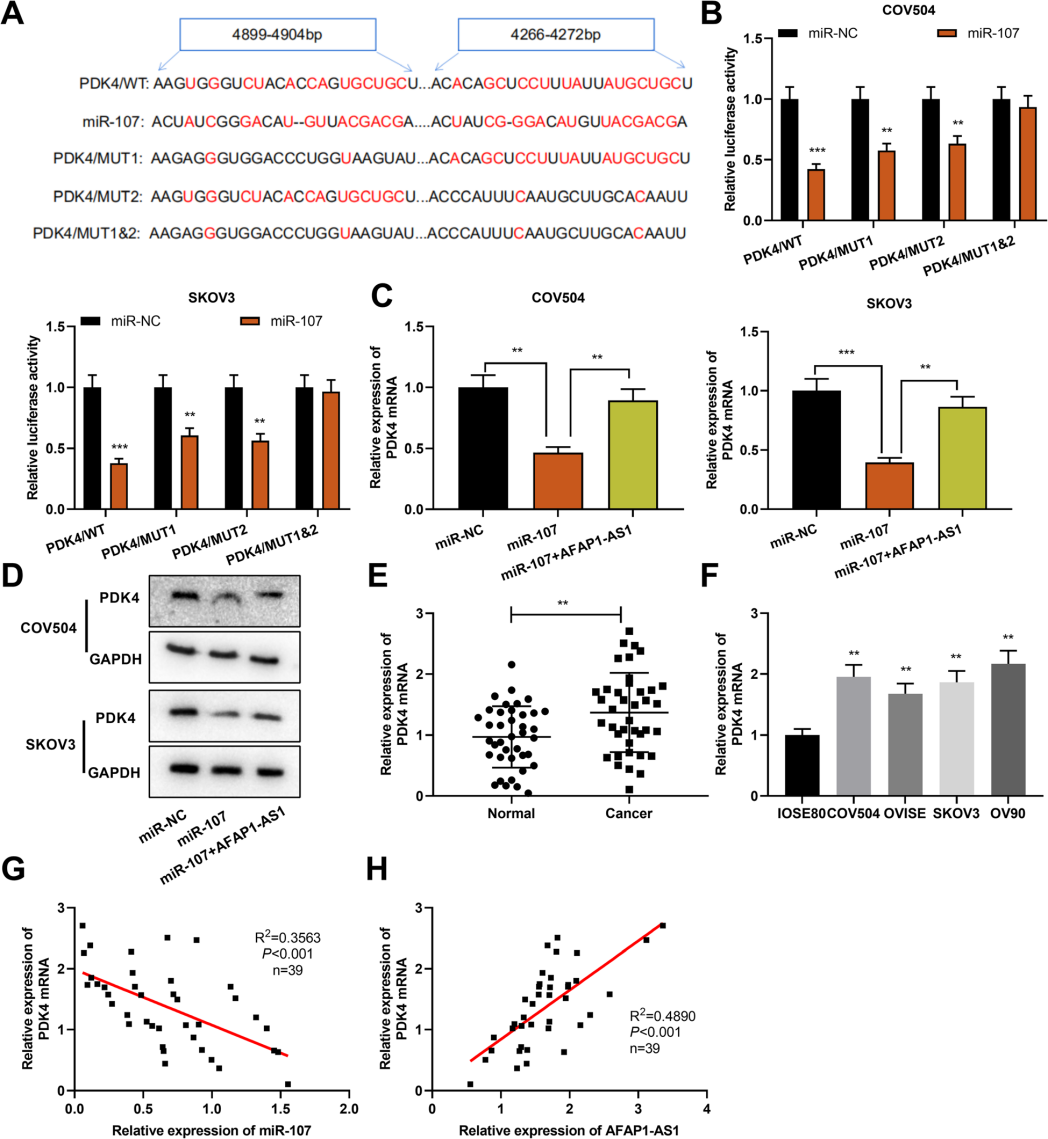
PDK4 was the target of miR-107. **a**. Bioinformatics analysis predicted the binding sequences between miR-107 and PDK4 3’UTR. **b**. Luciferase reporter gene assay was conducted for detecting the binding relationship between miR-107 and PDK4. **c & d**. The qRT-PCR and Western blot were used to detect the expression levels of PDK4 mRNA and protein in transfected OC cells. **e & f**. qRT-PCR was performed to detect PDK4 mRNA expression in OC tissues and cells. **g & h**. Correlation between PDK4 mRNA expression level and miR-107 expression level or AFAP1-AS1 expression level in OC tissues. **P < 0.01 and ***P < 0.001

To further study the potential mechanism of the AFAP1-AS1/miR-107 axis in the progression of OC, we explored the downstream targets of miR-107. The StarBase database was employed for screening the potential target genes of miR-107, and it was discovered that PDK4 was one of the potential targets of miR-107 (Fig. 5a). Subsequently, the targeted relationship between miR-107 and PDK4 was verified by the dual-luciferase reporter gene assay (Fig. 5b). Next, qRT-PCR and Western blot assays suggested that miR-107 remarkably repressed PDK4 mRNA and protein expression levels in COV504 and SKOV3 cells, while AFAP1-AS1 reversed this effect (Fig. 5c&d). In addition, PDK4 mRNA expression in OC was examined by qRT-PCR, and it was found that PDK4 mRNA expression in OC tissues and cells was dramatically enhanced as against para-cancerous tissues or IOSE80 cells (Fig. 5e&f), and in OC tissues, PDK4 mRNA expression was negatively correlated with miR-107 expression and positively correlated with AFAP1-AS1 expression (Fig. 5g&h). In summary, PDK4 was a downstream target of miR-107 in OC cells, and AFAP1-AS1 modulated PDK4 expression via competitively binding to miR-107.

### MiR-107 regulated OC cell proliferation, migration and invasion via targeting PDK4

**Fig. 6:**
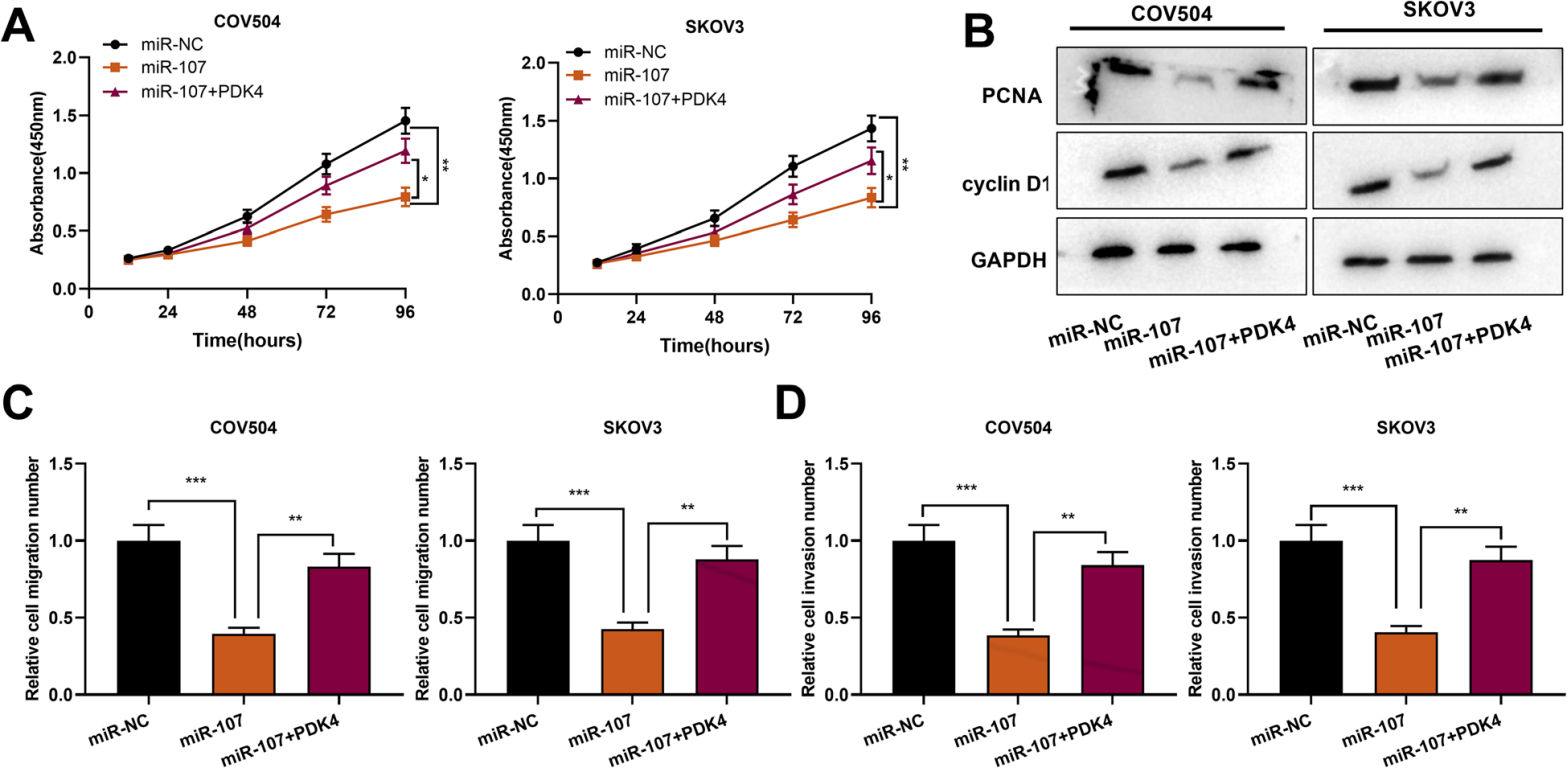
MiR-107 targeted PDK4 to inhibit OC cell proliferation, migration and invasion. **a**. MiR-107 mimic and PDK4 overexpression plasmids were co-transfected into OC cells, and CCK-8 assay was used for examining cell proliferation after transfection. **b**. Western blot was used to detect PCNA and cyclin D1 protein expression levels. **c & d**. Transwell assays were used to detect cell migration and invasion after transfection. *P < 0.05, **P < 0.01 and ***P < 0.001

We further investigated the functional roles of miR-107/PDK4 axis in OC cells. The results of CCK-8 assay showed that PDK4 overexpression counteracted miR-107 overexpression-mediated inhibiting effect on cell proliferation (Fig. 6a), and the inhibiting impact of miR-107 on PCNA and cyclin D1 expression levels was also reversed (Fig. 6b). In the Transwell assays, compared with the miR-107 group, the cell migration and invasion capacities were markedly enhanced in the miR-107 + PDK4 group (Fig. 6c&d). The above-mentioned evidence indicated that miR-107 repressed OC cell proliferation, migration and invasion via targeting PDK4.

## Discussion

Increasing evidence shows that lncRNA dysregulation is closely associated with OC occurrence and development, and lncRNAs are expected to become new markers for OC diagnosis and treatment [18]. A report shows that lncRNA FAM83H-AS1 is highly expressed in OC, and facilitates OC cell proliferation, metastasis and radioresistance by stabilizing HuR protein [19]. The up-regulation of lncRNA TP73-AS1 expression is related to the OC patient’s poor prognosis, and it can facilitate OC cell proliferation and metastasis by regulating MMP2 and MMP9 [20]. AFAP1-AS1 plays a cancer-promoting role in various cancers [21]. For example, AFAP1-AS1 is discovered to be significantly highly expressed in laryngeal cancer tissues, and by regulating the miR-320a/RBPJ axis, AFAP1-AS1 enhances the cancer cells’ stemness and resistance to chemotherapy [22]. This research reported that AFAP1-AS1 was highly expressed in OC cells and tissues, and its expression was correlated with FIGO stage. It was also revealed that AFAP1-AS1 knockdown dramatically repressed OC cell proliferation, migration and invasion, and down-regulated PCNA and cyclin D1 expression levels in OC cells. The findings displayed that AFAP1-AS1 was likely to facilitate OC progression. A previous study also reports that AFAP1-AS1 promotes OC cell proliferation and inhibits the apoptosis [12]. However, our work further found that it also had an impact on cell migration and invasion.

Almost half of miRNAs are transcribed from cancer-associated genomic regions, which are often amplified, deleted or rearranged in tumors, featuring prominently in the tumorigenesis and progression of OC and other cancers [23]. For example, miR-126-3p can inhibit OC cell proliferation and invasion via targeting PLXNB2 [24]. In this study, miR-107, whose expression was markedly decreased in OC cells and tissues, was predicted and proved to be the direct target of AFAP1-AS1. We further confirmed that miR-107 inhibition reversed AFAP1-AS1 knockdown-caused inhibiting impact on OC cell proliferation, migration and invasion. These findings manifested that AFAP1-AS1 could modulate the progression of OC via sponging miR-107. It is reported that miR-107 can target CCNE1 to inhibit OC cell cycle progression and proliferation [17], which is consistent with our finding that miR-107 shows the tumor-suppressing effects. Similarly, miR-107 expression is reduced in breast cancer, and miR-107 inhibits breast cancer cell proliferation and migration via targeting and suppressing HMGB1 [25]. Conversely, miR-107 is significantly elevated in colorectal cancer tissues and cell lines, and it inhibit SW480 and LoVo cell apoptosis by targeting PAR4 [26]. The distinct roles of miR-107 may be resulted from the characteristics of its different target genes in different cancers.

Known as a member of the PDK family, PDK4 is a key enzyme implicated in glucose metabolism and mitochondrial respiratory control [27, 28]. PDK4 is primarily expressed in the skeletal muscle and heart, and it is pivotal in regulating the activity of pyruvate dehydrogenase complex [27, 28]. It is reported that high PDK4 expression indicates a worse prognosis of OC patients [29], and PDK4 activates the STAT3/AKT/NF-κB/IL-8 signal pathway to enhance the metastasis and glycolysis of OC cells, and to help cancer cells maintain stemness [30]. In this research, we discovered for the first time that AFAP1-AS1 regulated PDK4 expression through competitively binding with miR-107 in OC. Moreover, PDK4 could dramatically counteract the inhibiting impact of miR-107 on OC cell proliferation, migration and invasion. The above findings confirmed that AFAP1-AS1 indirectly elevated PDK4 expression by sponging miR-107, thus promoting OC progression.

## Conclusions

To sum up, the present work highlights that AFAP1-AS1 is a pivotal factor in OC progression. Moreover, for the first time, we report the regulatory relationship of AFAP1-AS1/miR-107/ PDK4 axis, and this is likely to help better understand OC pathogenesis.

## Data Availability

The data used to support the findings of this study are available from the corresponding author upon request.
